# Sleep and Microdialysis: An Experiment and a Systematic Review of Histamine and Several Amino Acids

**DOI:** 10.5334/jcr.183

**Published:** 2019-07-03

**Authors:** Cathalijn H. C. Leenaars, W. H. Pim Drinkenburg, Christ Nolten, Maurice Dematteis, Ruud N. J. M. A. Joosten, Matthijs G. P. Feenstra, Rob B. M. De Vries

**Affiliations:** 1 SYRCLE, Radboud Institute for Health Sciences, Radboud University Medical Center, NL; 2 Department of Animals in Science and Society – Human-Animal Relationship, Faculty of Veterinary Sciences, Utrecht University, NL; 3 Institute for Laboratory Animal Science, Hannover Medical School, DE; 4 Janssen Research and Development, a division of Janssen Pharmaceutica N.V., BE; 5 Department of Addiction Medicine, Grenobles Alpes University Hospital and Grenoble Alpes University, FR; 6 Netherlands Institute for Neuroscience (NIN), An Institute of the Royal Netherlands Academy of Arts and Sciences, NL

**Keywords:** Amino acids, Proline, Microdialysis, Prefrontal cortex, Sleep, Systematic Review, Glutamate, Glutamine, Glycine, Aspartate, Asparagine, GABA, Histamine, Taurine, Proline

## Abstract

Sleep seems essential to proper functioning of the prefrontal cortex (PFC). The role of different neurotransmitters has been studied, mainly the catecholamines and serotonin. Less attention has been paid to the amino acid transmitters and histamine. Here, we focus on the activity of these molecules in the PFC during sleep and sleep deprivation (SD). We determined extracellular concentrations of histamine and 8 amino acids in the medial PFC before, during and after SD. Additionally, we systematically reviewed the literature on studies reporting microdialysis measurements relating to sleep throughout the brain. In our experiment, median concentrations of glutamate were higher during SD than during baseline (p = 0.013) and higher during the dark-active than during the resting phase (p = 0.003). Glutamine was higher during post-SD recovery than during baseline (p = 0.010). For other compounds, no differences were observed between light and dark circadian phase, and between sleep deprivation, recovery and baseline. We retrieved 13 papers reporting on one or more of the molecules of interest during naturally occurring sleep, 2 during sleep deprivation and 2 during both. Only two studies targeted PFC. Histamine was low during sleep, but high during sleep deprivation and wakefulness, irrespective of brain area. Glu (k = 11) and GABA (k = 8) concentrations in different brain areas were reported to peak during sleep or wakefulness or to lack state-dependency. Aspartate, glycine, asparagine and taurine were less often studied (1-2 times), but peaked exclusively during sleep. Sleep deprivation increased glutamate and GABA exclusively in the cortex. Further studies are needed for drawing solid conclusions.

## Introduction

Sleep seems necessary for prefrontal cortex (PFC)-dependent tasks. Prefrontal regions show prominent deactivation during sleep [1] and sleep deprivation [2]. Besides, prefrontal tasks such as attention, working memory, temporal memory and behavioural inhibition are affected by sleep deprivation in humans [3], but also in animals, e.g. on tasks addressing working memory and task switching [4,5]. To learn more about the neurobiological mechanisms involved, extracellular concentrations of neurotransmitters have been studied. In contrast to monoamines [6] and adenosine [7], other transmitters that are important in sleep regulation [8] such as the amino acids and histamine (Hist) did not receive much attention. Therefore we focus here on 1) Hist, a monoamine that is as important as the other monoamines in the regulation of sleep and wakefulness but is much less studied, and 2) excitatory (e.g. glutamate (Glu) and aspartate (Asp)) and inhibitory (e.g. GABA (gamma-aminobutyric acid), glycine (Gly) and taurine (Tau)) neurotransmitters and neuromodulators [9,10].

Microdialysis is a convenient and elegant method to study extracellular biochemistry over time. Extracellular concentrations present direct information on the activity status of neurotransmitters and neuromodulators in a certain brain region, in contrast to total brain concentrations [11]. Microdialysis is based on simple diffusion through a semi-permeable membrane; from the tissue of interest into a continuously flowing isotonic fluid. The dialysate is then analysed by ultra-high-pressure liquid chromatography (UPLC).

In this paper we describe our experiment that investigates the effects of 12h of sleep deprivation on medial PFC (mPFC) concentrations of Hist, asparagine (Asn), Asp, GABA, Glu, glutamine (Gln), Gly, proline (Pro) and Tau, analysing left-over microdialysis sample volume from another experiment [12]. We selected these amino acids as they can be measured in a single UPLC run [13,14]. Because this experiment was a spin-off, we did not have clear *a priori* hypotheses on concentration changes, nor did we specifically design our experiment for measurements of amino acids. The experiment should be considered hypothesis-generating. Also, we did not analyse the literature on amino acids in detail upfront. To put our data into perspective, we did perform a systematic review (SR) of sleep and sleep deprivation studies measuring these molecules in animal brain microdialysates afterwards.

## Materials and Methods

### mPFC Microdialysis Experiment

Our experimental protocol has been described previously, with measurements of dialysate corticosterone [12], adenosine [7] and monoamine concentrations [6] before, during and after sleep deprivation. We now describe Hist and the amino acids Asn, Asp, GABA, Glu, Gln, Gly, Pro and Tau. We restricted our analysis to these molecules, as they can be measured in a single UPLC run. A comparable publication on monoamines measured in the same dialysates can be found in Menon et al. [6].

In short, male Wistar rats (N = 11, 250–300g) were prepared for microdialysis with custom-made (e.g. [15]) concentric microdialysis probes (the probe was implanted directly, without the use of a guide cannula; exposed membrane length: 4 mm), placed into the mPFC at an angle of 12° (AP + 3.0 mm; L ± 1.8 mm; V–5.5 mm; relative to Bregma) [16]. The experiment started after approximately 1 week of post-surgical recovery. Rats were placed into separate compartments of a sleep deprivation device after connection to the microdialysis set-up. Perfusion of artificial cerebrospinal fluid (145 mmol/l NaCl, 1.2 mmol/l CaCl_2_, 2.7 mmol/l KCl, 1.0 mmol/l MgCl_2_) was set at a flow rate of 3 μl/min. Sampling continued for 64h, comprising 12h of wash-out and simultaneous habituation to the experimental environment (19:00–07:00), 24h of baseline (07:00–07:00), 12h of sleep deprivation during the light phase (modelling one sleepless night in humans; (07:00–19:00) and 16h of recovery (19:00–11:00). Sleep deprivation was achieved using variable forced locomotion in an upright rotating drum, divided into 2 semi-circular compartments by a stationary central wall [12]. This method has previously been shown to suppress slow wave sleep to <1% and rapid eye movement (REM) sleep completely [12].

Rats were on a non-reversed 12:12 light-dark cycle without dusk/dawn transitions. Food and normal tap water were available ad libitum throughout the experiment. The experiment was approved by the experimental animal committee of the Royal Netherlands Academy of Arts and Sciences (NIN2006-03 & NIN2007-20). It was performed in accordance with national legislation and standards of care.

#### Microdialysates

Dialysate samples were collected for 1h periods (i.e. 180 μl) in plastic vials (300 μl; 7431100, Aurora Borealis) in a refrigerated fraction collector (6°C; CMA 470, Aurora Borealis). Samples were transferred to ice during the experiment and split into 8 fractions. The fractions destined for Hist and amino acid measurements were transported on dry ice from Amsterdam to Beerse, where they were again stored at –80°C until analysis. After the experiment, all fractions were stored at –80°C. Corticosterone [12], adenosine [7] and monoamines [6] were determined in other fractions.

Hist and amino acids were determined by Janssen Pharmaceutica, Research & Development (Beerse, Belgium), department of Neuroscience Systems Biology. The standard protocol comprises UPLC with fluorescent detection (UPLC-FD) after derivatization with 6-aminoquinolyl-N-hydroxy-succinimidyl-carbamate (AQC), as described before by Cohen et al. [13] and Liu et al. [14].

#### Primary Data-Analysis

Analyses of our experimental data were performed in Microsoft Excel and SPSS 24.0.0.0. In line with our analyses for monoamines [6] and adenosine [7], we selected analyses that limit the effects of outliers on the results. We thus report median values and the interquartile range (IQR) for each phase of the experiment, instead of the generally reported mean ± standard error.

The median of the 12h blocks for baseline–light, baseline–dark, sleep deprivation and recovery were determined for each animal. Differences between these phases were analysed with a non-parametric Friedman repeated-measures test for each analysed molecule separately. When the Friedman test indicated an overall significant difference, post-hoc Wilcoxon signed rank tests were used to compare the sleep deprivation and recovery periods with their corresponding baseline, and to compare baseline light phase with baseline dark phase. No other post-hoc tests were performed.

### Systematic Review

We previously published a systematic mapping review on Hist and the amino acids Asn, Asp, GABA, Glu, Gln, Gly, Pro and Tau as assessed by intracerebral microdialysis [17]. In this SR, we followed the same methodology up to the selection of papers.

#### Search

In brief, a comprehensive search for studies on Hist and the amino acids Asn, Asp, GABA, Glu, Gln, Gly, Pro and Tau retrieved relevant publications from two databases: PubMed and Embase. Searches were performed in 2016, on 12 December for PubMed and on 14 December for Embase. Duplicates were manually removed in Endnote.

Our previously developed search filter for microdialysis [18] was extended by adding the terms “chemitrode”, “dialytrode”, “brain dialysis”, “intracerebral dialysis”, “intracranial dialysis”, “transcranial dialysis” and “implanted perfused hollow fibre” to retrieve older microdialysis publications. Previously developed filters were used to select animal studies [19,20]. The complete search strategies for PubMed and EmBase are described in our protocol [21] and available in appendix 1.

#### Selection

Screening was performed in EROS (Early Review Organising Software; Institute of Clinical Effectiveness and Health Policy, Buenos Aires, Argentina) by two independent reviewers. Discrepancies were resolved by discussion among the reviewers. For our mapping review [17], we excluded (1) studies on other techniques than microdialysis (e.g. biosensors and microdialysis precursors such as push-pull perfusion), (2) studies measuring other substances than Hist and the amino acids Asn, Asp, GABA, Glu, Gln, Gly, Pro and Tau, (3) retro-dialysis studies, (4) microdialysis studies that did not report baseline values without the specified molecules in the perfusion fluid, (5) extra-cerebral microdialysis studies, (6) human and in vitro studies, and (7) papers not containing primary study data. Within publications, experiments using techniques other than microdialysis were also ignored (e.g. in [22]), as well as data on amino acids other than those we searched for (e.g. in [23]).

During screening, tags were added by KJ and CL to all studies on circadian rhythms, sleep and sleep deprivation. Tagged studies were subsequently screened for inclusion in this review based on the following criterion (besides being included in our mapping): studies measuring one or more of the molecules of interest during (1) naturally occurring sleep stages that were validated with polysomnographic measurements and/or (2) during sleep deprivation. Studies on e.g. carbachol-induced REM-sleep were thus excluded (e.g. in [23]).

To ensure capturing all relevant studies, we searched for studies with the terms “*sleep*”, “*REM*”, “*rest*”, “*fatig*”, and “*somn*” in the title within the studies included in our mapping review.

#### Data Extraction and Analysis

The following study characteristics were extracted for the included papers: bibliographic details, location of the laboratory, the number of animals (n); their species, strain, age, weight, and sex; the flow-rate, probe length, diameter, membrane, and reuse; brain region, wash-out, anaesthesia/freely behaving, post-surgical recovery, amino acids analysed, type of analysis, light-dark cycle and type of sleep deprivation. Furthermore, we extracted the study outcomes relevant to this review by summarising the findings, with the units of measurements as provided by the authors. For legibility, we here present the species, brain region and sleep deprivation method. The other data are provided in appendix 2–4. Data were tabulated separately for studies of naturally occurring sleep and for sleep deprivation studies.

#### Risk of Bias Assessment

Due to limited resources, only one experimenter (CL) estimated the risk of bias (RoB) of the included studies. For sleep deprivation studies, the standard tools of RoB assessment are only partially applicable [24], because many studies implement a within-subject design. Besides, risks of bias relating to blinding are usually high because it is virtually impossible to appropriately blind the experimenters for sleep deprivation. RoB assessment was thus adapted to comprise blinding of sample analysis for detection bias and incomplete outcome data for attrition bias [25]. Besides, we analysed two design aspects specific to microdialysis studies: verification of probe placement (to confirm that amino-acid changes were related to the brain region under investigation) and missing samples (decreased or interrupted flow due to a leakage or block can results in a number of missing measurements). Furthermore, we analysed other study quality aspects: reporting of an a priori power analysis, approval of an ethical board, and conflicts of interest.

## Results

### Experiment

Without missing samples, we would have collected 704 samples (64 samples of 1h in 11 rats). The fraction for amino acids was missing for 133 samples (19%, mostly due to minor fluctuations of the flow). Hist and the amino acids Asn, Asp, GABA, Glu, Gln, Gly, Pro and Tau were analysed in the remaining 571 samples.

The median values for wash-out, baseline light phase, baseline dark phase, 12h of sleep deprivation and 12h of recovery are provided in Table 1, with the corresponding interquartile range (IQR). The overall Friedman test was significant for several amino acids: Asn, Asp, Gln, Glu and Gly. For Asn, Asp and Gly, none of the post-hoc tests were significant. For these amino acids, the significance of the Friedman test is probably due to the concentrations being higher during wash-out than during the remainder of the experiment (the effect of wash-out was not post-hoc analysed, in line with our preceding analyses of adenosine [7] and monoamines [6]).

**Table 1:** Medial prefrontal cortex microdialysis results.

Amino acid	Phase	Median (μM)	25%–75% IQR (μM)	N	G(df)	p

Asn	Wash-out	1.28	0.40–2.24	10	20.3 (4)	<0.001
	Baseline Light	0.11	0.08–0.19	11		
	Baseline Dark	0.15	0.08–0.20	11		
	SD	0.11	0.10–0.20	11		
	Recovery	0.13	0.09–0.20	11		
	Sleep deprivation vs. Baseline Light: t = 42.5; p = 0.40					
	Recovery vs. Baseline Dark: t = 31.0; p = 0.86					
	Baseline Light vs. Baseline Dark: t = 40.0; p = 0.53					

Asp	Wash-out	0.06	0.05–0.08	10	20.3 (4)	<0.001
	Baseline Light	0.05	0.04–0.06	11		
	Baseline Dark	0.04	0.04–0.06	11		
	SD	0.05	0.04–0.06	11		
	Recovery	0.05	0.03–0.06	11		
	Sleep deprivation vs. Baseline Light: t = 42.5; p = 0.40					
	Recovery vs. Baseline Dark: t = 31.0; p = 0.86					
	Baseline Light vs. Baseline Dark: t = 40.0; p = 0.53					

GABA	Wash-out	0.09	0.07–0.13	10	3.44 (4)	0.49
	Baseline Light	0.09	0.06–0.13	11		
	Baseline Dark	0.10	0.05–0.11	11		
	SD	0.08	0.06–0.12	11		
	Recovery	0.09	0.06–0.11	11		

Gln	Wash-out	5.40	0.16–11.8	10	10.67 (4)	0.031
	Baseline Light	0.03	0.03–0.09	11		
	Baseline Dark	0.04	0.02–0.05	11		
	SD	0.07	0.02–0.09	11		
	Recovery	0.07	0.05–0.12	11		
	Sleep deprivation vs. Baseline Light: t = 31.0; p = 0.72					
	Recovery vs. Baseline Dark: t = 62.0; p = 0.010					
	Baseline Light vs. Baseline Dark: t = 23.0; p = 0.65					

Glu	Wash-out	0.10	0.53–1.44	10	15.2 (4)	0.004
	Baseline Light	0.16	0.09–0.19	11		
	Baseline Dark	0.31	0.25–0.34	11		
	SD	0.27	0.26–0.39	11		
	Recovery	0.34	0.20–0.50	11		
	Sleep deprivation vs. Baseline Light: t = 61.0; p = 0.013					
	Recovery vs. Baseline Dark: t = 46.0; p = 0.25					
	Baseline Light vs. Baseline Dark: t = 66.0; p = 0.003					

Gly	Wash-out	0.43	0.26–0.78	10	19.0 (4)	0.001
	Baseline Light	0.11	0.08–0.15	11		
	Baseline Dark	0.08	0.06–0.12	11		
	SD	0.08	0.06–0.11	11		
	Recovery	0.08	0.06–0.15	11		
	Sleep deprivation vs. Baseline Light: t = 3.0; p = 0.23					
	Recovery vs. Baseline Dark: t = 4.0; p = 0.55					
	Baseline Light vs. Baseline Dark: t = 4.0; p = 0.55					

Hist	Wash-out	0.04	0.03–0.05	10	3.1 (4)	0.54
	Baseline Light	0.03	0.03–0.05	10		
	Baseline Dark	0.04	0.03–0.05	11		
	SD	0.03	0.03–0.05	11		
	Recovery	0.03	0.03–0.04	11		

Pro	Wash-out	0.35	0.07–0.63	10	5.3 (4)	0.26
	Baseline Light	0.09	0.06–0.26	10		
	Baseline Dark	0.10	0.06–0.36	10		
	SD	0.12	0.07–0.30	11		
	Recovery	0.11	0.07–0.26	11		

Tau	Wash-out	0.61	0.55–0.85	10	3.8 (4)	0.44
	Baseline Light	0.88	0.51–1.14	11		
	Baseline Dark	0.85	0.60–1.14	11		
	SD	1.10	0.47–1.23	11		
	Recovery	0.60	0.35–0.97	11		

Asn = Asparagine, Asp = Aspartate, GABA = Gamma-AminoButyric Acid, Gln =Glutamine, Glu = Glutamate, Gly = Glycine, Hist = Histamine, Pro = Proline, Tau = Taurine, SD = Sleep Deprivation, IQR = InterQuartile Range, N = number of rats, G = the test statistic of the Friedman’s test, df = degrees of freedom, p = p-value of the Friedman’s test.

Median concentrations of Glu were higher during sleep deprivation than during corresponding baseline (p = 0.013). Besides, an effect of light-dark phase was observed, Glu concentrations were higher during the dark-active phase than during the rest phase (p = 0.003). Median concentrations of Gln were higher during post-SD recovery than during the corresponding baseline (p = 0.010), none of the other post-hoc tests were significant. No differences in median concentrations were observed between any of the test phases for GABA, His, Pro and Tau.

### Systematic Review

#### Search and Selection

Our original searches retrieved 5118 references from PubMed and 5253 from Embase. Screening started with the titles and abstracts of 6239 references after duplicate removal. Of these, 3144 were subsequently screened full-text. During full text screening, 47 references were tagged as relevant for circadian rhythms, sleep and sleep deprivation. Of these, 17 met the inclusion criterion for the current systematic review on sleep. Our search for relevant title words did not result in new references meeting the inclusion criteria.

**Figure 1:**
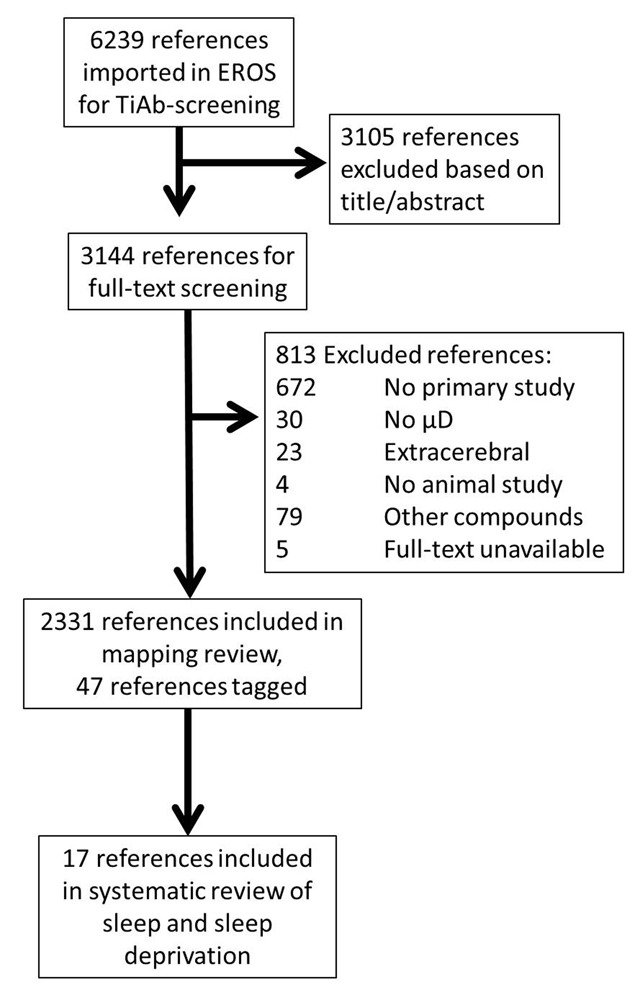
Flow scheme of retrieved and included references. TiAb – Title-Abstract; μD = microdialysis.

The flow of papers is shown in Figure 1.

#### Study Characteristics

The 17 included references describe measurements of the molecules of interest in cats (k = 7) and rats (k = 10; k = 2 Wistar, k = 8 Sprague-Dawley) in different brain regions. All included references were in English. Four references did not mention the sex of the animals, the others all used male animals only. The light schedule was adequately described by 9 out of 17 references. For these 9 references, the cycle was normal (12:12, lights on at 6:00 or 8:00) for 7, reversed for 1 (12:12, lights on at 18:00), and one used continuous light.

Microdialysis flow rate was reported in all references and ranged from 0.4 to 3 μl/min. Wash-out durations were reported by 13 out of 17 references and ranged from 45 minutes to 24h. Probe lengths were also reported in all references and ranged from 1 to 4 mm. Probe diameters were reported by 15 out of 16 references and ranged from 0.2 to 0.5 mm. Information on membrane type (e.g. Hospal or CMA7) and/or molecular weight cut off (1–50 kDa) was reported in 15 out of 17 references. None of the references described probe reuse.

The methods used for analysis comprised high performance liquid chromatography (HPLC) with fluorescence detection (k = 4), HPLC with electrochemical detection (k = 7), HPLC without a description of the detection method (k = 1), liquid chromatography (k = 1), capillary electrophoresis (k = 2) and radioenzymatic essay (k = 1). One reference did not describe the method used for sample analysis. Measurements comprised Hist only (k = 3), Glu only (k = 4), GABA only (k = 3), or several amino acids (k = 7).

Study characteristics are provided by study and amino acid in appendix tables 2 (for natural sleep studies) and 3 (for sleep deprivation studies).

#### Microdialysate Histamine and Amino Acids during naturally occurring Sleep

We retrieved 15 studies analysing one of the molecules of interest during naturally occurring sleep stages. The results are summarised in Table 2. Extracellular concentrations of amino acids during natural sleep stages were reported in 13 studies. Our search retrieved no studies of Pro. One study reported no state-dependency at all [26]. In all other studies one of more of the other amino acids except Gln showed state-dependent changes.

**Table 2:** Studies of microdialysate histamine and amino acid levels during naturally occurring sleep.

Study_ID	Species	Brain Area	Summary

Azuma_1996 [27]	Rats	mPOA	Glu was higher during wake (2.04 ± 0.22μM) and wake-to NREM transitions (2.22 ± 0.63μM) than during NREM sleep (1.56 ± 0.27μM) or NREM-to wake transitions (1.26 ± 0.28μM).
Chu_2004 [28]	Rats	Frontal cortex	Hist release was 3.8 times higher during wake episodes (0.19 ± 0.01 pmol/20 min) than during sleep episodes (0.05 ± 0.002 pmol/20 min).
Gronli_2007 [26]	Rats	Hippocampus	GABA levels did not show state-dependent variations (wake: 1600.5 ± 393.4 fmol/sample; SWS: 1530.5 ± 408.4 fmol/24μl sample; REM: 1503.0 ± 388.1 fmol/sample).
Hasegawa_2000 [29]	Rats	PRN	Gly levels (in the low μM range, exact values not provided) were higher during REM than during NREM and wake. Glu and Gln levels (in the low μM range, exact values not provided) were not state-dependent.
John_2008 [22]	Rats	TMN	No significant differences in hypothalamic Glu levels were observed between REM (2.5 ± 0.17 pmol/10μl sample), SWS (2.4 ± 0.06 pmol/sample) and W (2.5 ± 0.17; 2.4 ± 0.06 and 2.3 ± 0.12 pmol/sample).
Kekesi_1997 [23]	Cats	VPL	Asp, Asn, Glu, Gly, Tau and GABA were significantly elevated during SWS (exact values not provided) compared to the awake baseline (Asp: 1.7 ± 0.03 μM; Asn: 1.5 ± 2.0 μM; Glu: 6.6 ± 0.3 μM; Gly: 8.7 ± 0.6 μM; Tau: 17.8 ± 0.7 μM; GABA: 0.7 ± 0.2 μM). Gln did not show state-dependent variations (61.3 ± 3.4 μM at baseline wake).
Kodama_1998 [30]	Cats	PIA, NMC, NPM	Glu levels during SWS were taken as reference (NMC: 217.5 ± 21.9; PIA: 164.3 ± 18.1; NPM: 239.6 ± 23.5 fmol/min sample). During REMS, Glu in NMC increased to 111% of SWS, significantly different from Glu during W (98% of SWS). In the other areas no such difference was observed.
Lena_2005 [31]	Rats	mPFC, Nacc	In the NAcc, Glu was lower during SWS (5.69 × 10^–7^ ± 1.53 M) and REM (5.57 × 10^–7^ ± 1.47 M) than during wake (9.28 × 10^–7^ ± 2.89 M). ANOVA indicated a difference in Asp concentrations (SWS: 2.24 × 10^–7^ ± 1.3 M; REM: 2.25 × 10^–7^ ± 1.21 M; wake: 5.1 × 10^–7^ ± 2.3 M), but post-hoc analyses were negative. No state-dependent changes in Asp and Glu levels were observed in the mPFC (wake Asp: 2.51 × 10^–7^ ± 0.18 M; Glu: 16.2 × 10^–7^ ± 2.9 M; SWS Asp: 2.79 × 10^–7^ ± 0.58 M; Glu: 16.1 × 10^–7^ ± 6 2.5 M; REM Asp: 2.33 × 10^–7^ ± 0.28 M; Glu: 15.9 × 10^–7^ ± 2.6 M).
LopezRodriguez_2007 [32]	Rats	Orbitofrontal Cortex	Glu levels (range: 0.17–5.28 μM) were higher during REM (134 ± 14% of overall mean) than during wake (106 ± 5%), and lower during SWS (87 ± 5%) than during wake.
Nitz_1997a [33]	Cats	Dorsal raphe	GABA was higher during REM sleep (0.072 ± 0.003 pmol/μL) than during SWS (0.049 ± 0.007 pmol/μL) and wake (0.042 ± 0.005 pmol/μL). Glu (wake: 35.84 ± 2.55 pmol/μL; SWS: 36.42 ± 4.57 pmol/μL; REM: 33.98 ± 2.03 pmol/μL) and Gly (wake: 32.30 ± 4.29 pmol/μL; SWS: 34.05 ± 4.80 pmol/μL; REM: 33.89 ± 4.11 pmol/μL) levels were not state-dependent.
Nitz_1997b [34]	Cats	Locus Coeruleus	GABA was higher during REM sleep (1.91 ± 0.2 fmol/μL) than during SWS (1.58 ± 0.2 fmol/μL) and wake (1.2 ± 0.3 fmol/μL). Glu (wake: 0.88 ± 0.20 pmol/μL; SWS: 0.90 ± 0.20 pmol/μL; REM: 0.95 ± 0.25 pmol/μL) and Gly (wake: 0.54 ± 0.16 pmol/μL; SWS: 0.45 ± 0.14 pmol/μL; REM: 0.48 ± 0.13 pmol/μL) levels were not state-dependent.
Strecker_2002 [35]	Cats	POAH	Hist was highest during wakefulness (1.155 ± 0.225 pg/μl), lower during SWS (0.395 ± 0.081 pg/μl) and lowest during REM-sleep (0.245 ± 0.032 pg/μl). Note that after conversion (wake: 0.312 ± 0.061 pmol/20 min; REM 0.066 ± 0.009; SWS 0.107 ± 0.022), these values are quite comparable to those of Chu [28].
Vanini_2011 [36]	Cats	PRN	GABA decreased during REM (0.268 ± 0.050 pmol/10μL) versus both wake (0.466 ± 0.046 pmol/10μL) and SWS (0.365 ± 0.046 pmol/10μL), which did not differ from each other.
Vanini_2012 [37]	Cats	sCTX, BF	In the BF, GABA levels were higher during SWS (1.042 pmol/10μL) than during REM (0.836 pmol/10μL) and wake (0.977 pmol/10μL). In the somatosensory cortex, GABA levels were higher during SWS (1.275 pmol/10μL) than during wake (1.15 pmol/10μL) and REM (0.932 pmol/10μL).
Watson_2011 [38]	Rats	PRN	Concentrations of both Glu and GABA were higher during wake (Glu: 1.948 ± 0.018 μM; GABA: 0.428 ± 0.003 μM) than during NREM (Glu 85.8% of wake; GABA 89.0%) and REM (Glu 83.6% of wake; GABA 81.3%). Asp (0.522 ± 0.003 μM during wake), Tau (7.442 ± 0.055 μM during wake) and Gly (4.942 ± 0.046 μM during wake) did not show state-dependent changes.

ANOVA = ANalysis Of VAriance; BF = Basal Forebrain; mPFC = Medial PreFrontal Cortex; NAcc = Nucleus Accumbens; NMC = Nucleus MagnoCellularis; NPM = Nucleus ParaMedianus; PIA = Pontine Inhibitory Area; POAH = Preoptic/Anterior Hypothalamic Area; NREM = Non-REM; PRN = Pontine Reticular Formation; sCTX = somatosensory Cortex; SWS = Slow Wave Sleep; TMN = TuberoMammillary Nucleus; VPL = VentroPosteroLateral thalamic nuclei.

Mixed results were reported for most amino acids, with at least one study also showing negative findings (Glu, GABA, Asp, Gly, Tau). Dependent on the study and the target area, concentrations of Glu and GABA, in particular, peaked during either wakefulness or one of the sleep stages, suggesting that the differences were possibly region-specific. Gly, Asp and Asn were only reported to peak during a sleep stage or to be not state-dependent.

Two studies reported Hist concentrations (Table 2). The findings were consistent, as both observed higher concentrations during wakefulness than during sleep.

#### Microdialysate Histamine and Amino Acids during Sleep Deprivation

We retrieved five studies analysing one of the molecules of interest during sleep deprivation; two on Hist, one on Glu, one on GABA and one on Glu and GABA. The results are summarised in Table 3. Similar results were reported for Glu and GABA, increased extracellular concentrations in cortex and hippocampus, but no change in another area. Hist concentrations during SD were similar to those in wakefulness and higher than during sleep, irrespective of the area studied. Our search retrieved no sleep deprivation studies of the amino acids Asn, Asp, Gln, Gly, Pro or Tau.

**Table 3:** Sleep deprivation studies.

Study_ID	Species	Brain Area	SD method	Summary

John_2008 [22]	Rats	hypothalamus, cortex	Gentle Handling	Sleep deprivation had no significant effect on hypothalamic Glu (2.8 ± 0.02 vs. 2.5 ± 0.04 pmol/sample), but it increased cortical Glu (0.72 ± 0.14 μM vs. 0.35 ± 0.05 μM).
Strecker_2002 [35]	Cats	POAH	Gentle Handling	Hist levels during sleep deprivation (no exact values given) were comparable with those during wakefulness (1.155 ± 0.225 pg/μl; after conversion: 0.312 ± 0.061 pmol/20 min).
Vanini_2012 [37]	Cats	CTX, BF	na	Cortical GABA increased during extended wakefulness, BF GABA levels did not (no exact values given).
Xie_2015 [39]	Rats	Hippocampus	Platform-water tank (PSD)	Glu and GABA were higher at the end of 24h of paradoxical sleep deprivation (Glu: 500 ± 138 μmol/L; GABA: no exact values given) than during baseline (Glu: 0.74 ± 0.07 μmol/L; GABA: 0.27 ± 0.03 μmol/L). Of note, microdialysis flow was decreased during the SD period. Increased recovery due to decreased flow rate may have confounded these findings.
Zant_2012 [40]	Rats	Basal Forebrain	Gentle Handling	Sleep deprivation increased Hist levels (in the nM range, no exact values given).

BF = Basal Forebrain; CTX = Cortex; na = information not available; POAH = Preoptic/Anterior Hypothalamic Area.

#### Risk of Bias and study quality

Assessment of bias due to study design features and other quality aspects were analysed for all references together (sleep and sleep deprivation studies), by reference, and are shown in appendix 4. None of the 17 included references described blinding of sample analysis, which could result in detection bias, particularly when chromatography peaks are manually integrated. Only two out of 17 described missing samples, which could result in attrition bias. Only 2 out of 17 contained a statement describing the absence of conflicts of interest. Of note, for the studies of amino acids during naturally occurring sleep, the performed analyses were not always appropriately conducted; dependence of the data of included animals was regularly ignored (e.g. [27]).

None of the 17 included references described power analyses, which could result in underpowered studies. Approval of the protocol by an ethical board was reported in 10 out of 17 references. Methods for verification of probe placement were reported in 15 out of 17 references, of which 10 showed the results.

## Discussion

Our systematic review shows that differences in extracellular brain concentrations of the major amino acid neurotransmitters Glu and GABA between sleep stages have repeatedly been reported, but do not show regular patterns. At this moment, no specific conclusions may be drawn, except that state-dependency of Glu and GABA seems to be brain region specific. Even less can be concluded from the reports on the other amino acids (Gly, Asp, Tau, Gln, Asn, Pro) and effects of SD, as published data are scarce and often conflicting. Hist, on the other hand, consistently shows similar peak concentrations during wakefulness and sleep deprivation, and lower levels during sleep stages. The results are consistent with the general organization and function of these neurotransmitters: the monoamine Hist providing a general modulatory influence throughout the brain originating in a small histaminergic nucleus; the neurotransmitter amino acids providing direct excitatory or inhibitory signals organized in numerous different neuroanatomical pathways.

The results of our experiment are a case in point, as the data presented in Tables 1, 2, 3 and in our previous review of brain amino acids and circadian rhythms [17] are conflicting. We found state-dependency of prefrontal extracellular Glu, consistent with our preceding review of circadian rhythms of Glu [24], which shows higher levels of Glu during the dark than during the light phase in several brain regions. However, our observation of increased Glu and Gln during or after SD is not in line with the results presented for actual sleep/wake stages (Table 2), which are much more diverse or negative. Only one sleep deprivation study included in our review analysed cortical Glu, which increased [29], consistent with our experimental findings. The positive results for Glu results are corroborated by sensor-based measurements of cortical extracellular Glu with high temporal resolution, showing Glu peaks during wake and REM sleep and during SD [41]. The apparent conflict may be resolved if we look at the brain areas of these two sets of studies. Except for 2 studies in the nucleus accumbens [31,42], which report higher Glu during the active circadian phase and during wakefulness, the lists of brain regions do not show any overlap. The tentative conclusion is that Glu is differently related to sleep/wake stages in different brain regions.

We did not observe any significant effect of sleep stages or SD on the concentrations of the other amino acids we studied. Comparison of the data in Table 2, the data in the previous review [17] and our present experimental data supports the above-mentioned lack of consistent findings and possible importance of regional differences. Asp e.g. was found to be high during the active phase in the suprachiasmatic nucleus, but in other areas higher in slow wave sleep (SWS) than wake (Table 2) or not state-dependent (Tables 1 and 2). In our experiment, median concentrations of Gln were higher during post-SD recovery than during the corresponding baseline. However, no evidence for increased Gln during any sleep/wake stage or circadian phase was found.

Neither did we find any alteration in Hist levels. These experimental results are discordant with the preceding literature for circadian rhythms concerning Hist, where increased levels were observed in the frontal cortex and other areas during the dark phase compared to the light phase in rodents and cats [17,28,35,43,44]. They do not agree either with the conclusion from our present review on specific sleep/wake stages. Our failure to replicate the consistent pattern described previously is difficult to explain. We note that our experimental set-up and methodology differed in various ways from what was used in the other studies, and that the concentrations that we report (0.03 – 0.04 μmol/L) are 3–10 times higher than the previously reported concentrations [28,35,40], but we cannot be certain that these differences can explain our neutral findings.

Of note, our experiment was not specifically designed for analyses of amino acids; we used a relatively large probe (4 mm) and high flow rate (3 μl/min) compared to most of the studies included in the review (Appendix table 2). While we did not properly analyse washout effects, our results are indicative of them for Asn, Asp and Gly; the overall Friedman test was significant, and median concentrations were higher during wash-out than during any of the other phases. Differences in the experimental design (flow rate, probe size, duration, etc.) may result in different functional changes of the brain tissue, which could potentially explain inconsistent findings between experiments.

Several systematised reviews on the microdialysis technique have been published, but we are aware of only two of them on amino acids [17,45]. Fliegel et al. performed meta-analyses on Glu and GABA concentrations in relation to ethanol exposure [45]. We combined a mapping of all studies describing baseline dialysate concentrations of Hist and the amino acids Asn, Asp, GABA, Glu, Gln, Gly, Pro and Tau with a systematic review of the circadian rhythms of these molecules [17].

Tagging and/or labelling of references on specific topics during screening for large reviews is a potentially efficient approach to increase the output and relevance of review efforts. In the here-described review, early-stage labelling for individual amino acids was combined with tagging for circadian rhythms and sleep. Labelling concordance for amino acids ranged from 93.6% to 100% in a subset of 140 papers analysed by two screeners [17]. Our tagging for studies on sleep was very successful, our title searches of all references included in our preceding mapping review [17] did not result in any additional references on sleep. We still advise against labelling and tagging for 2 topics simultaneously for future reviews, and furthermore against labelling for complex concepts (e.g. analyses of circadian rhythmicity, where our tagging approach was less successful).

The overall risk of bias of the included studies is unclear, and reporting quality is at most reasonable. Reporting of experimental detail was poor, although reporting of the microdialysis technique was relatively good. Of specific concern for the studies comparing sleep stages between subjects is the relatively high prevalence of suboptimal statistical analyses; most studies comparing amino acid levels between sleep stages selected individual sleep episodes as the unit of measurement, ignoring the dependency of the data and artificially inflating the statistical power resulting from small numbers of animals included. Fortunately, some of the studies did use the individual animal as the unit of measurement, and specific statistical analyses for within subject comparisons (e.g. [31]).

To conclude, data on amino acid concentrations in the mPFC in relation to sleep are still scarce, and further primary studies are warranted. Systematic reviews are still relatively uncommon in preclinical sleep science and neurochemistry. With our approach, we hope to inspire neurochemists, chronobiologists and sleep scientists to implement more systematised review strategies.

## Data Accessibility Statement

Data in this publication have not yet been made publicly available. Provided data from the microdialysis experiment are sufficient for inclusion in future meta-analyses. Data included in the systematic review are already in the public domain. Full data will be made available for reuse to individual scientists upon reasonable requests.

## Additional Files

The additional files for this article can be found as follows:

10.5334/jcr.183.s1 Appendix 1. Search.

10.5334/jcr.183.s2 Appendix 2. Study characteristics of sleep studies.

10.5334/jcr.183.s3 Appendix 3. Study characteristics of sleep deprivation studies.

10.5334/jcr.183.s4 Appendix 4. Risk of Bias and study quality.

## References

[1] Hobson, JA and Pace-Schott, EF. The cognitive neuroscience of sleep: neuronal systems, consciousness and learning. Nat Rev Neurosci. 2002; 3: 679–693. DOI: 10.1038/nrn915 12209117

[2] Thomas, M, Sing, H, Belenky, G, Holcomb, H, Mayberg, H, Dannals, R, Wagner, H, Thorne, D, Popp, K, Rowland, L, et al. Neural basis of alertness and cognitive performance impairments during sleepiness. I. Effects of 24 h of sleep deprivation on waking human regional brain activity. J Sleep Res. 2000; 9: 335–352. DOI: 10.1046/j.1365-2869.2000.00225.x 11123521

[3] Muzur, A, Pace-Schott, EF and Hobson, JA. The prefrontal cortex in sleep. Trends Cogn Sci. 2002; 6: 475–481. DOI: 10.1016/S1364-6613(02)01992-7 12457899

[4] Leenaars, CH, Joosten, RN, Zwart, A, Sandberg, H, Ruimschotel, E, Hanegraaf, MA, Dematteis, M, Feenstra, MG and van Someren, EJ. Switch-task performance in rats is disturbed by 12 h of sleep deprivation but not by 12 h of sleep fragmentation. Sleep. 2012; 35: 211–221. DOI: 10.5665/sleep.1624 22294811 PMC3250360

[5] Reichert, CF, Maire, M, Schmidt, C and Cajochen, C. Sleep-Wake Regulation and Its Impact on Working Memory Performance: The Role of Adenosine. Biology (Basel). 2016; 5 DOI: 10.3390/biology5010011 PMC4810168 26861410

[6] Menon, JML, Nolten, C, Achterberg, EJM, Joosten, RNJMA, Dematteis, M, Feenstra, MGP, Drinkenburg, WH and Leenaars, CHC. Brain Microdialysate Monoamines in Relation to Circadian Rhythms, Sleep, and Sleep Deprivation – a Systematic Review, Network Meta-analysis, and New Primary Data. J Circadian Rhythms. 2019; 17: 1 DOI: 10.5334/jcr.174 30671123 PMC6337052

[7] Leenaars, CHC, Savelyev, SA, Van der Mierden, S, Joosten, R, Dematteis, M, Porkka-Heiskanen, T and Feenstra, MGP. Intracerebral Adenosine During Sleep Deprivation: A Meta-Analysis and New Experimental Data. J Circadian Rhythms. 2018; 16: 11 DOI: 10.5334/jcr.171 30483348 PMC6196573

[8] Scammell, TE, Arrigoni, E and Lipton, JO. Neural Circuitry of Wakefulness and Sleep. Neuron. 2017; 93: 747–765. DOI: 10.1016/j.neuron.2017.01.014 28231463 PMC5325713

[9] Cooper, JR, Bloom, FE and Roth, RH. The Biochemical Basis of Neuropharmacology. New York: Oxford University Press; 2003.

[10] Feldman, RS, Meyer, JS and Quenzer, LF. Principles of Neuropsychopharmacology. Sunderland, Massachusetts: Sinauer Associates, Inc; 1997.

[11] Westerink, BH, Damsma, G, Rollema, H, De Vries, JB and Horn, AS. Scope and limitations of in vivo brain dialysis: a comparison of its application to various neurotransmitter systems. Life Sci. 1987; 41: 1763–1776. DOI: 10.1016/0024-3205(87)90695-3 2889121

[12] Leenaars, CH, Dematteis, M, Joosten, RN, Eggels, L, Sandberg, H, Schirris, M, Feenstra, MG and Van Someren, EJ. A new automated method for rat sleep deprivation with minimal confounding effects on corticosterone and locomotor activity. J Neurosci Methods. 2011; 196: 107–117. DOI: 10.1016/j.jneumeth.2011.01.014 21262261

[13] Cohen, SA and Michaud, DP. Synthesis of a fluorescent derivatizing reagent, 6-aminoquinolyl-N-hydroxysuccinimidyl carbamate, and its application for the analysis of hydrolysate amino acids via high-performance liquid chromatography. Anal Biochem. 1993; 211: 279–287. DOI: 10.1006/abio.1993.1270 8317704

[14] Liu, H, Sanuda-Pena, MC, Harvey-White, JD, Kalra, S and Cohen, SA. Determination of submicromolar concentrations of neurotransmitter amino acids by fluorescence detection using a modification of the 6-aminoquinolyl-N-hydroxysuccinimidyl carbamate method for amino acid analysis. J Chromatogr A. 1998; 828: 383–395. DOI: 10.1016/S0021-9673(98)00836-X 9916319

[15] Feenstra, MG and Botterblom, MH. Rapid sampling of extracellular dopamine in the rat prefrontal cortex during food consumption, handling and exposure to novelty. Brain Res. 1996; 742: 17–24. DOI: 10.1016/S0006-8993(96)00945-6 9117391

[16] Paxinos, G and Watson, CJ. The Rat Brain in Stereotaxic Coordinates. Cambridge, Massachusetts: Academic Press; 2004.

[17] Leenaars, CHC, Freymann, J, Jakobs, K, Menon, JML, Van Ee, TJ, Elzinga, J, Kempkes, RWM, Zoer, B and Drinkenburg, P. A Systematic Search and Mapping Review of Studies on Intracerebral Microdialysis of Amino Acids, and Systematized Review of Studies on Circadian Rhythms. J Circadian Rhythms. 2018; 16: 12 DOI: 10.5334/jcr.172 30483349 PMC6196574

[18] van der Mierden, S, Savelyev, SA, IntHout, J, de Vries, RBM and Leenaars, CHC. Intracerebral microdialysis of adenosine and adenosine monophosphate – a systematic review and meta-regression analysis of baseline concentrations. J Neurochem. 2018; 147: 58–70. DOI: 10.1111/jnc.14552 30025168 PMC6220825

[19] Hooijmans, CR, Tillema, A, Leenaars, M and Ritskes-Hoitinga, M. Enhancing search efficiency by means of a search filter for finding all studies on animal experimentation in PubMed. Lab Anim. 2010; 44: 170–175. DOI: 10.1258/la.2010.009117 20551243 PMC3104815

[20] de Vries, RB, Hooijmans, CR, Tillema, A, Leenaars, M and Ritskes-Hoitinga, M. A search filter for increasing the retrieval of animal studies in Embase. Lab Anim. 2011; 45: 268–270. DOI: 10.1258/la.2011.011056 21890653 PMC3175570

[21] Leenaars, CHC, Van Luijk, JAKR, Freymann, J, Van Ee, TJ, Zoer, B, Drinkenburg, WH and De Vries, RBM. Amino acids in microdialysates (protocol). vol. 2018 www.SYRCLE.nl; 2017.

[22] John, J, Ramanathan, L and Siegel, JM. Rapid changes in glutamate levels in the posterior hypothalamus across sleep-wake states in freely behaving rats. Am J Physiol Regul Integr Comp Physiol. 2008; 295: R2041–2049. DOI: 10.1152/ajpregu.90541.2008 18815208 PMC2685298

[23] Kekesi, KA, Dobolyi, A, Salfay, O, Nyitrai, G and Juhasz, G. Slow wave sleep is accompanied by release of certain amino acids in the thalamus of cats. Neuroreport. 1997; 8: 1183–1186. DOI: 10.1097/00001756-199703240-00025 9175110

[24] Pires, GN, Bezerra, AG, Tufik, S and Andersen, ML. Effects of experimental sleep deprivation on anxiety-like behavior in animal research: Systematic review and meta-analysis. Neurosci Biobehav Rev. 2016; 68: 575–589. DOI: 10.1016/j.neubiorev.2016.06.028 27345144

[25] Hooijmans, CR, Rovers, MM, de Vries, RB, Leenaars, M, Ritskes-Hoitinga, M and Langendam, MW. SYRCLE’s risk of bias tool for animal studies. BMC Med Res Methodol. 2014; 14: 43 DOI: 10.1186/1471-2288-14-43 24667063 PMC4230647

[26] Gronli, J, Fiske, E, Murison, R, Bjorvatn, B, Sorensen, E, Ursin, R and Portas, CM. Extracellular levels of serotonin and GABA in the hippocampus after chronic mild stress in rats. A microdialysis study in an animal model of depression. Behav Brain Res. 2007; 181: 42–51. DOI: 10.1016/j.bbr.2007.03.018 17477980

[27] Azuma, S, Kodama, T, Honda, K and Inoue, S. State-dependent changes of extracellular glutamate in the medial preoptic area in freely behaving rats. Neurosci Lett. 1996; 214: 179–182. DOI: 10.1016/0304-3940(96)12918-9 8878113

[28] Chu, M, Huang, ZL, Qu, WM, Eguchi, N, Yao, MH and Urade, Y. Extracellular histamine level in the frontal cortex is positively correlated with the amount of wakefulness in rats. Neurosci Res. 2004; 417–420. DOI: 10.1016/j.neures.2004.05.001 15236867

[29] Hasegawa, T, Azum, S and Inoue, S. Amino acid release from the rat oral pontine reticular nucleus across the sleep-wakefulness cycle. J Med Dent Sci. 2000; 47: 87–93. 12162531

[30] Kodama, T, Lai, YY and Siegel, JM. Enhanced glutamate release during REM sleep in the rostromedial medulla as measured by in vivo microdialysis. Brain Res. 1998; 780: 178–181. DOI: 10.1016/S0006-8993(97)01308-5 9497097 PMC8848830

[31] Lena, I, Parrot, S, Deschaux, O, Muffat-Joly, S, Sauvinet, V, Renaud, B, Suaud-Chagny, MF and Gottesmann, C. Variations in extracellular levels of dopamine, noradrenaline, glutamate, and aspartate across the sleep–wake cycle in the medial prefrontal cortex and nucleus accumbens of freely moving rats. J Neurosci Res. 2005; 81: 891–899. DOI: 10.1002/jnr.20602 16041801

[32] Lopez-Rodriguez, F, Medina-Ceja, L, Wilson, CL, Jhung, D and Morales-Villagran, A. Changes in Extracellular Glutamate Levels in Rat Orbitofrontal Cortex During Sleep and Wakefulness. Archives of Medical Research. 2007; 52–55. DOI: 10.1016/j.arcmed.2006.07.004 17174723

[33] Nitz, D and Siegel, J. GABA release in the dorsal raphe nucleus: role in the control of REM sleep. Am J Physiol. 1997; 273: R451–455. DOI: 10.1152/ajpregu.1997.273.1.R451 9249585 PMC8855516

[34] Nitz, D and Siegel, JM. GABA release in the locus coeruleus as a function of sleep/wake state. Neuroscience. 1997; 78: 795–801. DOI: 10.1016/S0306-4522(96)00549-0 9153658 PMC8852367

[35] Strecker, RE, Nalwalk, J, Dauphin, LJ, Thakkar, MM, Chen, Y, Ramesh, V, Hough, LB and McCarley, RW. Extracellular histamine levels in the feline preoptic/anterior hypothalamic area during natural sleep-wakefulness and prolonged wakefulness: an in vivo microdialysis study. Neuroscience. 2002; 113: 663–670. DOI: 10.1016/S0306-4522(02)00158-6 12150786

[36] Vanini, G, Wathen, BL, Lydic, R and Baghdoyan, HA. Endogenous GABA levels in the pontine reticular formation are greater during wakefulness than during rapid eye movement sleep. J Neurosci. 2011; 31: 2649–2656. DOI: 10.1523/JNEUROSCI.5674-10.2011 21325533 PMC3073841

[37] Vanini, G, Lydic, R and Baghdoyan, HA. GABA-to-ACh ratio in basal forebrain and cerebral cortex varies significantly during sleep. Sleep. 2012; 35: 1325–1334. DOI: 10.5665/sleep.2106 23024430 PMC3443758

[38] Watson, CJ, Lydic, R and Baghdoyan, HA. Sleep duration varies as a function of glutamate and GABA in rat pontine reticular formation. J Neurochem. 2011; 118: 571–580. DOI: 10.1111/j.1471-4159.2011.07350.x 21679185 PMC3144159

[39] Xie, F, Li, X, Bao, M, Shi, R, Yue, Y, Guan, Y and Wang, Y. Anesthetic propofol normalized the increased release of glutamate and gamma-amino butyric acid in hippocampus after paradoxical sleep deprivation in rats. Neurol Res. 2015; 37: 1102–1107. DOI: 10.1080/01616412.2015.1114231 26923580

[40] Zant, JC, Rozov, S, Wigren, HK, Panula, P and Porkka-Heiskanen, T. Histamine release in the basal forebrain mediates cortical activation through cholinergic neurons. J Neurosci. 2012; 13244–13254. DOI: 10.1523/JNEUROSCI.5933-11.2012 22993440 PMC6621481

[41] Dash, MB, Douglas, CL, Vyazovskiy, VV, Cirelli, C and Tononi, G. Long-term homeostasis of extracellular glutamate in the rat cerebral cortex across sleep and waking states. J Neurosci. 2009; 29: 620–629. DOI: 10.1523/JNEUROSCI.5486-08.2009 19158289 PMC2770705

[42] Castaneda, TR, de Prado, BM, Prieto, D and Mora F. Circadian rhythms of dopamine, glutamate and GABA in the striatum and nucleus accumbens of the awake rat: modulation by light. J Pineal Res. 2004; 177–185. DOI: 10.1046/j.1600-079X.2003.00114.x 15009508

[43] Fell, MJ, Flik, G, Dijkman, U, Folgering, JH, Perry, KW, Johnson, BJ, Westerink, BH and Svensson, KA. Glutamatergic regulation of brain histamine neurons: In vivo microdialysis and electrophysiology studies in the rat. Neuropharmacology. 2015; 1–8. DOI: 10.1016/j.neuropharm.2015.05.034 26100446

[44] Hong, ZY, Huang, ZL, Qu, WM and Eguchi, N. Orexin A promotes histamine, but not norepinephrine or serotonin, release in frontal cortex of mice. Acta Pharmacol Sin. 2005; 155–159. DOI: 10.1111/j.1745-7254.2005.00523.x 15663891

[45] Fliegel, S, Brand, I, Spanagel, R and Noori, HR. Ethanol-induced alterations of amino acids measured by in vivo microdialysis in rats: a meta-analysis. In Silico Pharmacol. 2013; 1: 7 DOI: 10.1186/2193-9616-1-7 25505652 PMC4230485

