# Supplementary material for: Sleep and Microdialysis: An Experiment and a Systematic Review of Histamine and Several Amino Acids
Source: J Circadian Rhythms. 2019 Jul 3;17:7. doi: 10.5334/jcr.183 (PMC6611484; doi:10.5334/jcr.183)
Supplement: Appendix 1. — Search. [file jcr-17-183-s1.pdf]

Appendix 1: Search

PubMed (Search performed: 12 December 2016)

Amino Acids
Amino Acids[MeSH] OR ((Amino[tiab] OR Aminergic[tiab]) AND (Acid[tiab]OR Acids[tiab])) OR
Asparagine
Asparagine[MeSH] OR Asparagine*[tiab] OR Asn[tiab] OR (carbamoylpropanoic AND (Acid[tiab] OR Acids[tiab])) OR
Aspartate
Aspartic Acid[MeSH] OR Aspart*[tiab] OR ((Aspar*[tiab] OR aminosuccinic[tiab]) AND (Acid[tiab] OR Acids[tiab])) OR Asp[tiab] OR
GABA
gamma-Aminobutyric Acid[MeSH] OR GABA[tiab] OR ((Aminobut*[tiab] OR Butanoic[tiab] OR Butyric[tiab]) AND (Acid[tiab] OR Acids[tiab])) OR
Glutamate
Glutamic Acid[MeSH] OR Glutamate[tiab] OR Glu[tiab] OR ((Glutamic[tiab]OR Glutaric[tiab]OR Aminopentanedioic[tiab]) AND (Acid[tiab] OR Acids[tiab])) OR
Glutamine
Glutamine[MeSH] OR Glutamine[tiab] OR Gln[tiab] OR (carbamoylbutanoic[tiab] AND (Acid[tiab] OR Acids[tiab])) OR
Glycine
Glycine[MeSH] OR Glycine*[tiab] OR Gly[tiab] OR (Aminoacetic[tiab] AND (Acid[tiab] OR Acids[tiab])) OR glycocoll[tiab] OR
Histamine
Histamine[MeSH] OR Histamine [tiab] OR His[tiab] OR ethanamine[tiab] OR Histidine[tiab] OR
Proline
Proline[MeSH] OR Proline[tiab] OR Pro[tiab] OR
Taurine
Taurine[MeSH] OR Taurine[tiab] OR Tau[tiab] OR ((Tauric[tiab] OR aminoethanesulfonic[tiab] OR aminoethane[tiab]) AND (Acid[tiab] OR Acids[tiab])) AND

Microdialysis
microdialysis[MeSH] OR micro dial*[tiab] OR microdial*[tiab] OR microD[tiab] OR chemitrode[tiab] OR dialytrode[tiab] OR brain dialys*[tiab] OR intracerebral dialys*[tiab] OR cerebral dialys*[tiab] OR intracranial dialys*[tiab] OR cranial dialys*[tiab] OR transcranial dialys*[tiab]

AND the **SYRCLE animal filter** (Hooijmans, C.R., et al., Enhancing search efficiency by means of a search filter for finding all studies on animal experimentation in PubMed. Laboratory animals, 2010. 44(3): p. 170-175.)

Embase (Search performed: 14 December 2016)

Amino Acids
amino Acid/ OR ((Amino OR Aminergic)AND (Acid OR Acids)).ti,ab,kw. OR
Asparagine
asparagine/ OR (Asparagine* OR Asn OR (carbamoylpropanoic AND (Acid OR Acids))).ti,ab,kw. OR
Aspartate
aspartic Acid/ OR (Aspart* OR ((Aspar* OR Aminosuccinic) AND (Acid OR Acids)) OR Asp).ti,ab,kw. OR
GABA
4 aminobutyric Acid/ OR GABA.ti,ab,kw. OR ((Aminobut* OR Butanoic OR Butyric) AND (Acid OR Acids)).ti,ab,kw. OR
Glutamate

glutamic Acid/ OR Glutamate.ti,ab,kw. OR Glu.ti,ab,kw. OR ((Glutamic OR Glutaric OR Aminopentanedioic) AND (Acid OR Acids)).ti,ab,kw. OR

Glutamine

glutamine/ OR (glutamine OR Gln OR (carbamoylbutanoic AND (Acid OR Acids))).ti,ab,kw. OR

Glycine

glycine/ OR (Glycine* OR Gly OR (Aminoacetic AND Acid) OR glycocoll).ti,ab,kw. OR

Histamine

histamine/ OR (Histamine OR His OR ethanamine OR Histidine).ti,ab,kw. OR

Proline

proline/ OR (Proline OR Pro).ti,ab,kw. OR

Taurine

taurine/ OR (Taurine OR Tau OR ((Tauric OR aminoethanesulfonic OR aminoethane) AND (Acid OR Acids))).ti,ab,kw. AND

Microdialysis

microdialysis/ OR (micro dial* OR microdial* OR microD OR chemitrode OR dialytrode OR brain dialys* OR intracerebral dialys* OR cerebral dialys* OR intracranial dialys* OR cranial dialys* OR transcranial dialys*).ti,ab,kw.

AND the **SYRCLE animal filter** (de Vries, R., et al., Updated version of the Embase search filter for animal studies. 2014.)
