# Supplementary material for: Sleep and Microdialysis: An Experiment and a Systematic Review of Histamine and Several Amino Acids
Source: J Circadian Rhythms. 2019 Jul 3;17:7. doi: 10.5334/jcr.183 (PMC6611484; doi:10.5334/jcr.183)
Supplement: Appendix 2. — Study characteristics of sleep studies. [file jcr-17-183-s2.pdf]

Appendix 2: Study characteristics of sleep studies

Study_ID	N	Strain	Age (weeks)	Weight (g)	Sex	Flow (µl/MIN)	Probe length (mm)	probe diameter (mm)	membrane	L/R	wash-out (hours)	post-surgical rec (days)	AA	Analysis	lights ON
Azuma_1996	4	Sprague-Dawley	8-9	300-310	m	2	2	0.22	CO 50 kDa	na	19	≥7	Glu	HPLC-FD	08:00-20:00
Chu_2004	4	Spraque-Dawley	8-11	260-320	m	2	3	0.5	CMA?	na	20	10	Hist	HPLC-FD	8:00 - 20:00
Gronli_2007	12	Spraque-Dawley	9	-300	m	1.2	4	0.5	CMA12	random	≥16	≥14	GABA	HPLC-ED	18:00-06:00
Hasegawa_2000	8	Spraque-Dawley	10-12	350-450	m	3	1	0.24	CMA11	na	~12	7-10	Gly, Glu, Gln	HPLC-ED	08:00-20:00
John_2008	na	Spraque-Dawley	na	300-400	m	2	1	0.22	AI-01 Eicom; CO 50kDa	na	~2	7	Glu	HPLC-FD	06:00-18:00
Kekesi_1997	na	na	na	3000-4000	na	1	3	0.2	Travenol; CO 5kDa	both	0,75	1	Asp, Asn, Glu, Gln, Gly, Tau, GABA	na	na
Kodama_1998	6	na	na	3000-4500	na	2	2	0.22	Eicom	na	12	na	Glu	HPLC-ED	na
Lena_205	7	Wistar	na	280-320	m	1	3 / 2	0.5	CMA12, CO 20kDa	left / right	4	14-21	Glu, Asp	Capillary Electrophoresis-FD	08:00-20:00
LopezRodriguez_2007	6	Spraque-Dawley	na	300-500	m	2	2	na	CMA11	na	24	7	Glu	HPLC-ED	06:00-18:00
Nitz_1997a	4	mongrel	adult	na	na	2	2	0.27	na		17	≥14	Glu, Gly, GABA	HPLC-ED	na
Nitz_1997b	4	mongrel	na	na	na	2	2	0.27	na	na	17	≥14	Glu, Gly, GABA	HPLC-ED	na
Strecker_2002	6	na	adult	na	m	1.5	2	0.5	CMA10, CO 20kDa	right	na	≥21	Hist	REA	24h
Vanini_2011	6	na	adult	na	m	3	2	0.5	CMA10 or CMA12, PAES, CO 20kDa	na	na	21-28	GABA	HPLC	na
Vanini_2012	3	na	adult	na	m	3	2	0.5	polycarbonate / polyarylether sulphone; CO 20kDa	Left / right	na	na	GABA	HPLC-ED	na
Watson_2011	10	Spraque-Dawley	adult	235-250	m	0.4	1	0.24	regenerated cellulose; CO 18kDa	na	na	17	Glu, GABA	capillary electroforesis - laser-induced FD	06:00-18:00
