# Supplementary material for: Sleep and Microdialysis: An Experiment and a Systematic Review of Histamine and Several Amino Acids
Source: J Circadian Rhythms. 2019 Jul 3;17:7. doi: 10.5334/jcr.183 (PMC6611484; doi:10.5334/jcr.183)
Supplement: Appendix 3. — Study characteristics of sleep deprivation studies. [file jcr-17-183-s3.pdf]

Appendix 3: Study characteristics of sleep deprivation studies

Study_ID	n	Strain	Age (weeks)	Weight (g)	Sex	Flow (µl/MIN)	Probe length (mm)	probe diameter (mm)	membrane	L/R	wash-out (hours)	post-surgical rec (days)	AA	Analysis	lights ON
John_2008	na	Spraque-Dawley	na	300-400	m	2	1	0.22	AI-01 Eicom; CO 50kDa	na	~2	7	Glu	HPLC-FD	06:00-18:00
Strecker_2002	6	na	na	na	m	1.5	2	0.5	CMA10, CO 20kDa	right	na	≥21	Hist	REA	24h
Vanini_2012	3	na	adult	na	m	3	2	0.5	Polycarbonate/ polyarylethersulphone, CO: 20kDa	Left / right	na	na	GABA	HPLC-ED	na
Xie_2015	16	Spraque-Dawley	na	250-300	m	2	2	0.5	CMA12, CO 20kDa	na	1	5	Glu, GABA	LC-FD	na
Zant_2012	8	Han-Wistar	12-16	300-400	m	1	2	0.24	CMA11	na	22-24	10	Hist	HPLC-FD	na
