# Supplementary material for: Sleep and Microdialysis: An Experiment and a Systematic Review of Histamine and Several Amino Acids
Source: J Circadian Rhythms. 2019 Jul 3;17:7. doi: 10.5334/jcr.183 (PMC6611484; doi:10.5334/jcr.183)
Supplement: Appendix 4. — Risk of Bias and study quality. [file jcr-17-183-s4.pdf]

Appendix 4: Risk of Bias and study quality

Study_ID	Blinding	missing samples	verification of probe placement	results of verification shown	power analysis	ethical review	conflict of interest
Azuma_1996	unclear	high (12.5%)	histology	no	NR	NR	NR
Chu_2004	unclear	unclear	NR	NR	NR	yes	NR
Gronli_2007	unclear	high (40%)	histology	no	NR	yes	NR
Hasegawa_2000	unclear	unclear	histology	yes	NR	NR	NR
John_2008	unclear	unclear	histology	no	NR	yes	NR
Kekesi_1997	unclear	unclear	NR	NR	NR	NR	NR
Kodama_1998	unclear	unclear	histology	yes	NR	NR	NR
Lena_2005	unclear	unclear	histology	yes	NR	NR	NR
LopezRodriguez_2007	unclear	unclear	histology	No	NR	yes	NR
Nitz_1997a	unclear	unclear	histology	yes	NR	NR	NR
Nitz_1997b	unclear	unclear	histology	yes	NR	NR	NR
Strecker_2002	unclear	unclear	histology	yes	NR	yes	NR
Vanini_2011	unclear	unclear	fluorescent microspheres	yes	NR	yes	NR
Vanini_2012	unclear	unclear	histology	yes	NR	yes	none
Watson_2011	unclear	unclear	histology	yes	NR	yes	none
Xie_2015	unclear	unclear	histology	no	NR	yes	NR
Zant_2012	unclear	unclear	histology	yes	NR	yes	NR

NR: Not Reported.
